# Evaluation of Protocol Biopsy Utility 12 Months after Renal Transplantation: A Multicenter Observational Analysis

**DOI:** 10.1155/2012/781263

**Published:** 2012-09-05

**Authors:** Bruno Moulin, Pierre Merville, Karine Renaudin, David Buob, Sophie Ferlicot, Michel Delahousse, Jacques Dantal, Laetitia Albano, Christelle Barbet, Georges Mourad, Laure-Hélène Noel

**Affiliations:** ^1^Service de Néphrologie-Transplantation, CHU Strasbourg, Hôpitaux Universitaires de Strasbourg, 1, place de l'Hôpital, 67091 Strasbourg, France; ^2^Service de Néphrologie-Transplantation-Dialyse, CHU Bordeaux, 33076 Bordeaux, France; ^3^Service d'Anatomie Pathologique, Centre Hospitalier Universitaire Hôtel Dieu, 44093 Nantes, France; ^4^Institut de Pathologie, CHRU de Lille and EA2686, Université Lille Nord de France, 59037 Lille, France; ^5^Service d'Anatomie et de Cytologie Pathologiques, Université Paris-Sud 11, APHP Hôpital de Bicêtre, 94270 Le Kremlin-Bicêtre, France; ^6^Service de Néphrologie et Transplantation, Foch Hospital, 92151 Suresnes, France; ^7^Institut de Transplantation, Urologie et Néphrologie CHU Nantes, 44093 Nantes, France; ^8^Service de Néphrologie et Transplantation Rénale, Hôpital Pasteur, 06002 Nice, France; ^9^Service de Néphrologie-Transplantation Rénale, Hôpital Bretonneau, 37044 Tours, France; ^10^Service de Néphrologie et Transplantation Rénale, Hôpital Lapeyronie, 34295 Montpellier, France; ^11^INSERM, Service de Transplantation, Service d'Anatomie Pathologique, Université René Descartes, Hôpital Necker, 75015 Paris, France

## Abstract

The clinical merit of surveillance kidney graft biopsies remains controversial. A retrospective, multicenter analysis evaluated 12-month surveillance biopsies (SB, 154 patients) versus no SB (NSB, 138 patients (11 with diagnostic biopsy)) in patients >18 months posttransplant with estimated GFR (eGFR) ≥30 mL/min. The primary objective was to describe renal function at 18 months post-transplant in patients with or without SB at month 12. Globally, most recipients in both cohorts were at low immunological risk (<10% of patients with PRA ≥30%). The immunosuppressive regimen remained unchanged following more than half of SB that exhibited chronic lesions (18/33, 54.5%). Mean (SD) eGFR at month 18 (primary endpoint) was 56 (19) mL/min/1.73 m² with SB and 54 (15) mL/min/1.73 m² with NSB (*P* = 0.48). In the SB group, slight nonspecific changes were observed in 51 cases, rejection (acute or chronic) in 6 cases, CNI-related toxicity in 15 cases, recurrence of initial disease in two cases, and interstitial fibrosis/tubular atrophy (IF/TA) in 83 cases (71.6%), of which 35 cases (30.2%) were grade II/III lesions. eGFR <50 mL/min/1.73 m² at month 6 predicted IF/TA grade II or III (OR 3.85, 95% CI 1.64, 9.05, *P* < 0.002). SB at 12 months posttransplant did not prompt significant modification of immunosuppression, and no renal benefit was observed.

## 1. Introduction

‘The mechanisms responsible for graft lesions are complex, involving multifactorial processes influenced by a range of donor and recipient factors as well as posttransplant events such as ischemia-perfusion injury, cellular and humoral immunity, viral and bacterial infections, and calcineurin inhibitor- (CNI-)related toxicity [1]. The Banff working classification of renal allograft pathology represents an internationally agreed, standardized classification of the morphologic changes observed on renal biopsy. The appearance of interstitial fibrosis/tubular atrophy (IF/TA), arteriolar hyalinosis, and arteriosclerosis usually precedes loss of kidney function [1], which becomes evident only at a relatively late stage of the process when the functional reserve is exhausted. Indeed, IF/TA is present in approximately half of all grafts with stable function by one year posttransplant [2].

Serum creatinine is the conventional marker for renal function, and while valuable for assessment of acute impairment of renal function (e.g., acute rejection) does not provide an accurate indication of renal tissue injury. Direct measurement of glomerular filtration rate (GFR) is more sensitive [3] but is costly and impractical in routine clinical care, while formulae that estimate GFR each have limitations and are vulnerable to potential bias [4–6], tending to underestimate the extent of function loss [7]. Early changes in proteinuria may be a more effective predictor of subsequent graft loss than estimated GFR (eGFR) [8–10], but again can only detect changes once lesions have progressed to the point where function is affected.

Against this background, some kidney transplant centers elect to perform surveillance biopsies (SB) in patients with stable graft function. One year posttransplant is a time point commonly used for performing such biopsies. The aim is to detect subclinical rejection or nonimmunological injury, notably CNI-related nephrotoxicity, and to quantify IF/TA [11]. However, SB are inconvenient for patients, carry a risk for complications [12], and incur additional cost [11]. Furthermore, robust evidence to confirm a benefit for SB in terms of graft function or survival is lacking, as is agreement on the optimal timing and frequency of biopsies. Accordingly, many centers choose only to perform a diagnostic biopsy in response to an overt clinical trigger such as an increase in serum creatinine coupled or not with rising proteinuria. The question of whether or not to undertake surveillance kidney biopsies remains controversial [11, 13].

A multicenter, retrospective analysis was undertaken to investigate the clinical utility of SB at one year after kidney transplantation. The aim of the analysis was to characterize the use of biopsies at several French transplant centers and assess the effect of 12-month SB on subsequent graft function. Renal function at 18 months posttransplant was compared between patients who did or did not undergo SB at one year. Characteristics of the biopsy procedures, histological findings, management responses, and risk factors or markers for IF/TA were also assessed.

## 2. Methods

### 2.1. Study Design and Objectives

This was a noninterventional, retrospective study undertaken in 17 kidney transplant centers in France from August 2007 to February 2010. Seven centers performed SB at 12 months posttransplantation, while ten centers performed only diagnostic biopsies with no surveillance biopsy (NSB). The primary objective was to describe renal function (based on eGFR) at 18 months after kidney transplantation in patients who did or did not undergo an SB at month 12 posttransplant. Secondary objectives included characterization of biopsy procedures, histological lesions identified on biopsy according to Banff 2005 classification, modifications of the immunosuppressive regimen made in response to biopsy findings, the change in renal function in relation to the presence or absence of IF/TA, and risk factors (notably histological) or markers for renal function impairment at 18 months posttransplantation.

### 2.2. Patients

Adult patients (≥18 years old) who had undergone kidney transplantation 18 months (±1 month) previously were eligible for inclusion if eGFR was ≥30 mL/min (aMDRD formula) at the time of study entry. For patients who underwent an SB at month 12 posttransplant, the biopsy was to have been performed at 12 months (±1 month). For patients who had undergone a diagnostic biopsy, this was to have been performed at 11–17 months posttransplant. Patients without biopsy during the period 11–18 months posttransplant were categorized as having had no biopsy.

All patients were required to have been treated with CNI and mycophenolic acid (MPA), with or without steroids, from the time of transplantation. For patients with a surveillance or diagnostic biopsy, this regimen was to have been continued from the initial posttransplant period to the time of the biopsy analyzed in the study and for patients with no biopsy until the point of inclusion in the study.

Key noninclusion criteria were multiorgan transplantation, presence of antidonor antibodies or panel reactive antibodies >80%, and treatment with azathioprine, an mTOR inhibitor or a molecule in development at any point between transplantation and the biopsy of the study, or between transplantation and inclusion in the study for the patients who had no biopsy.

### 2.3. Data Collection

Data were collected during a single routine clinic visit, based on medical records, questions and clinical examination. The following information were collected: (1) recipient and donor demographics, transplantation characteristics, previous infections and biopsy-proven acute rejection, and clinical events; (2) information on graft biopsy, including reasons for diagnostic biopsy, methods, quality, histology findings after central reading according to Banff 2005 classification [14]; and (3) local diagnosis based on surveillance or diagnostic biopsy results and any consequent changes to the immunosuppressive regimen; (4) renal function (serum creatinine, proteinuria) at months 3, 6, 12 (or day of the study biopsy), and month 18 (the day of inclusion to the study); (5) immunosuppressive agents used and dose, and blood concentration for CNIs. eGFR [aMDRD] was calculated *a posteriori* and was adjusted using a multiplying factor depending on dosage method of creatinine (all methods except colorimetric).

The most recent graft biopsy obtained prior to the data collection visit was analyzed centrally based on Banff 2005 criteria [14]. Four pathology experts evaluated all centrally read biopsy samples. Adequate quality was accepted for specimens with equal or more than 10 glomeruli and two arteries. Intermediate quality was retained for specimen with equal or more than seven glomeruli and one artery. Inadequate biopsy samples (less than seven glomeruli, and less than two vascular sections) were excluded from central analysis. C4d results were obtained locally.

### 2.4. Statistical Analysis

The sample size calculation indicated that 155 patients with SB and 138 patients without SB would be required to reach an absolute precision of ±3 mL/min/1.73 m² of the 95% confidence interval of eGFR at month 18 posttransplant in both groups assuming a standard deviation (SD) of 17 mL/min/1.73 m² and allowing for 20% of patients in the SB group and 10% in the NSB group being excluded due to inadequate biopsy samples (nQuery Advisor 4.0, Statistical Solutions, Saugus, MA, USA). Renal function parameters were compared between-groups using Students *t*-test or Wilcoxon signed-rank test. Other between groups comparisons were performed with the Chi squared, Fisher or Wilcoxon test. Factors associated with the presence of IF/TA grade II or III on univariate analysis (*P* < 0.1) were included as covariates in a multivariate logistic analysis. Statistical analyses were performed using SAS v8.2 (SAS Institute, Cary, NC, USA).

## 3. Results

### 3.1. Patients and Immunosuppression

A total of 292 patients were eligible for analysis, of whom 154 underwent a 12-month SB whereas there were 138 patients in the NSB group. Among the NSB patients, 127 had no biopsy, and 11 had a diagnostic biopsy. Baseline characteristics were similar in the groups of patients with SB or NSB other than a lower incidence of panel reactive antibodies in the range 31–80%, HLA incompatibilities, reduced use of induction therapy, and fewer patients with diabetes in the SB group (Table 1).

In both groups, patients received similar exposure to CNIs throughout the study. At month 12, the proportion of patients receiving cyclosporine was 26.6% (*n* = 41) and 26.8% (*n* = 37) in the SB and NSB groups, respectively (*P* = 0.97). Tacrolimus was administered in all remaining patients. The mean (SD) cyclosporine C_0_ concentration at month 12 was 123 (48) ng/mL in the SB group versus 131 (26) ng/mL (*P* = 0.52) in the NSB group. The corresponding mean C_2_ concentration was 773 (218) ng/mL versus 763 (195) ng/mL (*P* = 0.79). Tacrolimus trough concentration was 7.8 (2.4) ng/mL versus 8.3 (3.1) ng/mL (*n* = 0.14), respectively. The dose of MPA was lower in the SB group than in the NSB group at month 3 posttransplantation: 1158 (427) mg/day versus 1246 (381) mg/day based on enteric-coated MPA dosing (i.e., 1440 mg/day equivalent to mycophenolate mofetil [MMF] 2000 mg/day) (*P* = 0.048). MPA dose was similar at all other time points.

### 3.2. Renal Biopsies Evaluation (Local Examination)

In the SB group, the renal biopsies were performed at a mean of 12.2 (0.55) months after transplantation. Diagnostic biopsies were performed 12.8 (1.80) months after surgery for significant changes in renal function and/or proteinuria. Complications were rare, being observed in only 3.0% of all relevant biopsies, comprising macroscopic hematuria in three cases, arteriovenous fistula in two cases, and clotting of urinary ducts in one case.

In the SB group (*n* = 154), 93 (60.4%) and 32 cases (20.8%) were considered adequate or of intermediate quality for local pathological analysis. With local interpretation in the SB group, acute cellular rejection was identified in seven patients (5%), chronic rejection in 34 patients (22%), CNI-related nephrotoxicity in 19 patients (12%), and recurrence of initial nephropathy in two patients (1.3%). On the other hand, in the diagnostic biopsy group (*n* = 11), nonspecific IF/TA were seen in one patient (9.1%), CNI nephrotoxicity in five patients (45.5%), recurrence of initial disease in one patient (9.1%), and in four cases lesions were judged nonspecific, with no cases of acute rejection. No cases of positive C4d staining was reported in this group.

Following biopsy, the immunosuppressive regimen remained unchanged in the majority of cases (113/165, 68.5%) with no significant difference between groups (Table 2). No change was made to immunosuppressive regimen in more than half of all cases (54.5%) following detection of chronic lesions on SB. The most frequent modification following SB was to reduce or stop the dose of both the CNI and MPA, an approach that was followed in 15.4% of patients with no significant histological changes, 36.4% of patients with lesions classified as IF/TA, and 73.7% of patients with lesions classified as CNI-related toxicity. Otherwise, CNI-related toxicity prompted a reduction in the dose of CNI in only 2/5 diagnostic biopsies (40.0%).

The quality of biopsy material was estimated centrally according to Banff classification recommendations. 116 SB samples and eight diagnosis biopsy specimens were sent and considered adequate for interpretation.

### 3.3. Renal Function with or without Surveillance Biopsy

Mean values for the primary endpoint, eGFR (aMDRD) at month 18 posttransplant, were 56 (19) mL/min/1.73 m^2^ in the SB group and 54 (15) mL/min/1.73 m^2^ in the NSB group (*P* = 0.48). Nevertheless we observed a slight but significant difference in eGFR between SB and NSB groups at 3 and 6 months but not at 12 months (Table 3). There was no difference for eGFR between month 12 and month 18 for each group (56 mL/min/1.73 m^2^ at month 12 and month 18 in the SB group; 53 and 54 mL/min/1.73 m^2^, respectively, at month 12 and month 18 in the in the NSB group (no statistical test performed). Serum creatinine (not shown) and proteinuria were similar in both groups at months 12 and 18.

### 3.4. Histological Results of Central Reading of Surveillance Biopsies

Histological lesions from central reading of SB obtained 12 months after transplantation are summarized in Table 4. In 51 cases, renal biopsy showed no significant changes, corresponding to a near-normal kidney graft. Acute antibody-mediated rejection was not observed in any biopsy, while chronic antibody-mediated rejection was detected in only two cases (1.8%) with a positive-C4d staining. C4d-negative glomerulitis (g3) was observed in two patients (1.7%) and peritubular capillaritis (ptc3) in one patient (0.9%). Finally, acute and chronic cellular rejection was observed in two patients (1.8%) each. Significant IF/TA (i.e., grade ≥ II) was recognized in 35 cases (30.2%). Vascular lesions corresponding to fibrous intimal thickening and to arteriolar hyalinosis (not related to CNI-toxicity) were reported in 26 cases (22.4%) and 13 cases (11.2%), respectively. CNI toxicity was observed in 15 patients (12.9%). Other lesions included tubular necrosis in 12 patients (10.3%), thrombotic microangiopathy in 3 patients, (2.6%) and BK virus nephropathy in 1 patient (0.9%).

### 3.5. Relation between Renal Function and IF/TA on Central Biopsy Examination

**Figure 1:**
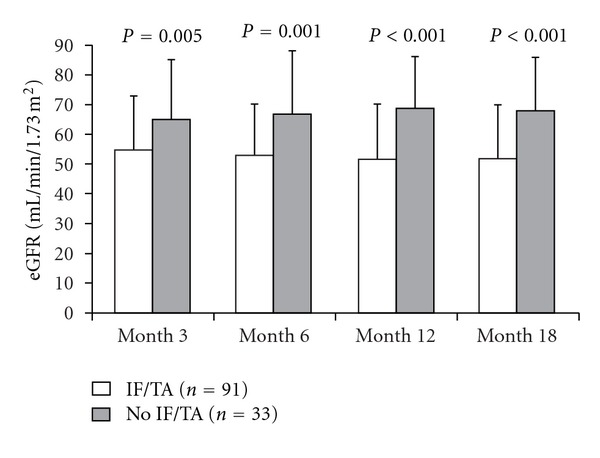
eGFR (aMDRD) according to the presence (*n* = 91) or absence (*n* = 33) of IF/TA detected on surveillance or diagnostic biopsy (mean ± SD).

In total, 83/116 (71.6%) SB and 8/8 (100.0%) diagnostic biopsies had IF/TA lesions (grade I to III). Nonspecific lesions with subnormal kidney were observed in 28.4% of screening biopsies and none of the diagnostic biopsies. In the SB group, grade I IF/TA was observed in 48 patients (41.4%), grade II in 25 patients (21.6%), and grade III in 10 patients (8.6%). Concerning the diagnostic biopsies, grade I was observed in five patients (62.5%), grade II in two patients (25%), and grade III in one patient (12.5%). Thus, grade II/III IF/TA lesions were detected in 35/116 (30.2%) of SB and 3/8 (37.5%) of diagnostic biopsies. Mean eGFR among patients with IF/TA detected either on surveillance or diagnostic biopsy (*n* = 91) was significantly different from that of patients without IF/TA on biopsy (*n* = 33) at all time points: month 3, 55  ±  18 versus  65  ±  20 (*P* = 0.005); month 6, 53 ± 17 versus 67 ± 21 (*P* = 0.001); month 12, 52 ± 18 versus 69 ± 17 (*P* < 0.001) and month 18, 52 ± 18 versus 68 ± 18 mL/min/1.73 m^2^ (*P* < 0.001) (Figure 1).

### 3.6. Risk Factors and Markers for IF/TA

On univariate analysis, diabetes, cold ischemia time ≥12 hours, delayed graft function, and eGFR <50 mL/min/1.73 m^2^ at month 3 or month 6 posttransplant all showed a significant association with detection of IF/TA grade II or III on central biopsy readings (Table 5). Multivariate analysis showed that eGFR <50 mL/min/1.73 m^2^ at month 6, diabetes, and cold ischemia time ≥12 h were associated with IF/TA grade II or III (Table 5).

## 4. Discussion

Kidney graft outcome is usually evaluated based on renal function, proteinuria and, in some centers, with protocol renal biopsies performed at varying times after transplantation. The value of protocol renal biopsies after kidney transplantation remains controversial. In this study, we evaluated the clinical utility of systematic SB performed at one year, using eGFR at 18 months posttransplant as the primary endpoint. Our results showed that renal function did not differ at 18 months after renal transplantation in patients with or without SB, but it should be borne in mind that the follow-up period after SB was relatively short.

This multicenter series of SB has confirmed the high incidence of IF/TA one year after kidney transplantation. Central reading of biopsies detected significant (i.e., grade ≥ II) IF/TA lesions in more than 30% of SB. Proteinuria, possibly a more sensitive marker of renal lesions [8–10], also showed no difference between the SB and NSB groups after month 3. The lack of renal benefit in the SB group is not surprising since immunosuppression was not modified following biopsy results. These results may suggest that histological data from SB performed at one-year might not unequivocally facilitate clinical management.

In total, the immunosuppressive regimen was amended in 52 patients (31.5%) after either a surveillance or diagnostic biopsy. Amendment of the regimen in approximately one third of patients is certainly valuable, but a greater response rate could be beneficial. This may be especially true in patients who exhibit histological evidence of CNI-related toxicity and IF/TA; here, 26.3% and 54.5% of such patients, respectively, remained under an unchanged regimen. The absence of immunosuppression modification (54.5%) in response to a local diagnosis of IF/TA reflects uncertainty about how to manage lesions of this type, and possibly the belief that such histological changes are irreversible. Decision-making is particularly challenging in view of the multifactorial etiology of IF/TA [15] and uncertainty as to whether histological damage becomes irreversible by one year [2]. In the absence of an accepted strategy to alter immunosuppression or other risk factors in response to the presence of IF/TA, the clinical value of SB is lessened. However, it was disappointing to observe that a quarter of patients received no reduction in CNI exposure following a diagnosis of immunosuppression-related toxicity. Here, the rationale to act is more clear-cut, and lessening of CNI dosage or introduction of an mTOR inhibitor is safe and may be beneficial [16].

The incidence of acute cellular rejection is usually maximal during the first months after transplantation, which may explain why central reading detected only two cases of acute cellular rejection in the SB group at one year (1.8%). Our findings are comparable with those of a recent randomized study in which the incidence of BPAR in kidney transplant patients receiving a regimen of CNI and MPA, with or without steroids, was only 4.8% during the period from 4.5 months to three years posttransplant [17]. The observed one-year incidence of BK virus nephropathy on SB (0.9%) was also similar to that reported in a large cohort of *de novo* kidney transplant patients receiving CNI and MPA with or without steroids within a recent randomized trial [18].

Diabetes, delayed graft function, and extended cold ischemia time showed a significant association with IF/TA on univariate analysis, with diabetes and cold ischemia time retaining a significant association on logistic regression analysis, as reported elsewhere [19, 20]. eGFR at month 6 using the aMDRD formula was predictive for IF/TA at one year posttransplant on both univariate and multivariate analysis. Values below 50 mL/min/1.73 m^2^ at month 6 were associated with a 3.85-fold increase in risk for moderate-to-severe IF/TA. Earlier eGFR values (month 3 posttransplant) showed no significant association with IF/TA. Other authors have reported a significant association between eGFR and IF/TA when analyzed concurrently. A recent subanalysis of data from 121 patients taking part in the CONCEPT study found a significant association between eGFR and the percentage of IF on SB, both at one year [21], a finding confirmed by other authors [22]. In a series of biopsies from 120 kidney-pancreas patients over a 10-year period, however, Nankivell et al. found the severity of IF/TA to be underestimated by measured GFR [2], a discrepancy that would be exacerbated by the variation between estimated and measured values of GFR [4–6]. Thus, while eGFR may have some predictive value, it cannot be regarded as a definitive indication of the presence or absence of IF/TA lesions.

The rate of complications arising from SB was low (2.6%), similar to findings in other series [12, 23], reflecting the reduction in risk associated with biopsy performed under ultrasound guidance (>70% of our patients). It is of concern, however, that only 60% of SB samples were considered adequate. Moreover, 42% of samples were considered normal on local analysis while central analysis of available biopsies observed significant IF/TA lesions in 49%. These results demonstrate that performing protocol graft biopsy at one year after kidney transplantation has no effect on renal function six months later, and is associated with only minimal modification of immunosuppressive therapy. Nevertheless, we agree that the delay is relatively short to evaluate the impact of this strategy.

The limitations of this analysis should be taken into account. As a retrospective study, selection bias is inevitably a concern. Sensitized patients (those with antidonor antibodies or panel reactive antibodies >80%) were excluded because of an increased propensity for diagnostic biopsies, and patients treated with azathioprine or an mTOR inhibitor were excluded in an attempt to homogenize the cohort. It is notable that renal function was significantly different between the SB and NSB groups at month 3 and month 6. It is not clear why such a difference existed, particularly since panel reactive antibodies and diabetes were more frequent in the NSB group. Neither is it clear why renal function remained stable during months 3 to 12 in the SB group but improved in the NSB group, such that values for eGFR became similar at month 12, when SB was undertaken. Secondly, the population was at low immunologic risk although almost one in five patients was positive for panel reactive antibodies. The findings from this cohort cannot necessarily be extrapolated to a higher-risk population. Thirdly, low rate of intervention in response to IF/TA lesions on SB in more than half of all cases—and no change to immunosuppression on detection of immunosuppression-related toxicity in a quarter of cases—undermined the potential benefits of SB in terms of improving renal function. Finally, and perhaps most importantly, the interval between surveillance biopsies and renal function assessment (6 months) was relatively short, and longer-term followup of this cohort, for example to at least two years posttransplant but ideally to four years, would be informative.

In conclusion, undertaking SB at one year after kidney transplantation was not associated with improved renal function at 18 months posttransplant in this retrospective analysis. SB results did not appear to influence immunosuppressive decision-making, such that the inconvenience, risk and cost of the SB program do not appear to have been justified. Importantly, the lack of renal benefit of one-year SB suggests that the informative lesions that might trigger adaptation of immunosuppression may instead be obtained from earlier biopsies. Longer followup is required, however. Additionally, prospective studies with protocol-specified modifications to the immunosuppressive regimen in response to specific histological diagnoses are required to determine whether routine biopsy of kidney transplant patients offers a long-term benefit for graft function, and to determine the optimal timing and frequency of biopsy. Notably, examination of the impact of SB at three months after kidney transplantation could be beneficial since earlier awareness of IF/TA and CNI-related nephrotoxicity could prompt immunosuppressive changes to protect the graft before irreversible damage is inflicted.

## Figures and Tables

**Table 1:** Baseline characteristics.

	Surveillance biopsy (*n* = 154)	No surveillance biopsy (*n* = 138)	*P* value
Male recipient, *n* (%)	90 (58.4%)	83 (60.1%)	0.77^a^
Recipient age, mean (SD), years	49.6 (13.5)	49.1 (12.6)	0.82^b^
Indication for transplantation, *n* (%)			
Glomerulonephritis	47 (30.5)	32 (23.2)	
Polycystic disease	30 (19.5)	20 (14.5)	
Hypertension/nephrosclerosis	16 (10.4)	6 (4.3)	NS
Diabetes	9 (5.8)	16 (11.6)	
Interstitial nephritis	5 (3.2)	9 (6.5)	
Other	22 (14.3)	35 (25.4)	
Unknown	25 (16.2)	20 (14.5)	
Previous kidney transplant, *n* (%)			
1	18 (11.7)	15 (10.9)	0.58^c^
≥2	1 (0.6)	3 (2.2)	
Panel reactive antibodies, *n* (%)			
1–30%	23 (15.6)	11 (8.1)	0.012^b^
31–80%	4 (2.7)	13 (9.6)	
Number of HLA incompatibilities, mean (SD)	3.5 (1.29)	3.9 (1.42)	0.002^b^
Old-to-old transplant, *n* (%)^d^	29 (18.8)	26 (18.8)	0.12^b^
Living donor, *n* (%)^b^	10 (6.5)	9 (6.5)	NS
Cold ischemia time (hours)			
Mean (SD)	16.3 (6.8)	17.5 (7.1)	0.09^a^
Induction therapy, *n* (%)			
Any	123 (79.9)	127 (92.0)	
IL-2 receptor antibody	66 (42.9)	59 (42.8)	0.003^b^
Antithymocyte globulin	47 (30.5)	51 (37.0)	
Other	9 (5.8)	15 (10.9)	
Delayed graft function, *n* (%)	30 (19.5)	29 (21.0)	0.74^b^
Hypertension, *n* (%)	131 (85.1)	106 (76.8)	0.07^b^
Dyslipidemia, *n* (%)	71(46.1)	66 (47.8)	0.77^b^
Diabetes, *n* (%)	13 (8.4)	24 (17.4)	0.022^b^

^a^Chi squared; ^b^Wilcoxon; ^c^Fisher; ^d^Both donor and recipient ≥60 years. NS: not significant.

**Table 2:** Modification of immunosuppression following biopsy and local diagnosis of normal, IF/TA or immunosuppression-related toxicity. All differences were nonsignificant.

	Surveillance biopsy (*n* = 154)	Diagnostic biopsy (*n* = 11)
All biopsies, *n* (%)		
No change	106 (68.8)	7 (63.6)
Continue CNI & MPA, both at reduced doses	32 (20.8)	3 (27.3)
Continue CNI & MPA with reduced CNI dose	2 (1.3)	1 (9.1)
Switch from CNI to another therapeutic class	3 (1.9)	0
Switch from MPA to another therapeutic class	2 (1.3)	0
Other	9 (5.8)	0
Normal, *n* (%)	*N* = 65	*N* = 1
No change	55 (84.6)	1 (100.0)
Continue CNI & MPA, both at reduced doses	10 (15.4)	0
IF/TA, *n* (%)	*N* = 33	*N* = 1
No change	18 (54.5)	0
Continue CNI & MPA, both at reduced doses	9 (27.3)	1 (100.0)
Switch from CNI to another therapeutic class	2 (6.1)	0
Switch from MPA to another therapeutic class	1 (3.0)	0
Other	3 (9.1)	0
CNI-related toxicity, *n* (%)	*N* = 19	*N* = 5
No change	5 (26.3)	3 (60.0)
Continue CNI & MPA, both at reduced doses	11 (57.9)	2 (40.0)
Continue CNI & MPA with reduced CNI dose	2 (10.5)	0
Switch from CNI to another therapeutic class	1 (5.3)	0

IF/TA: interstitial fibrosis/tubular atrophy; CNI: calcineurin inhibitor; MPA: mycophenolic acid.

**Table 3:** Renal function according to time posttransplant.

	Surveillance biopsy (*n* = 154)	No surveillance biopsy (*n* = 138)	*P* value^a^
eGFR, mL/min/1.73 m^2 b^			
Month 3	54 (20–107)	49 (15–114)	0.002
Month 6	55 (21–119)	52 (8–104)	0.025
Month 12	55 (16–138)	49 (25–99)	0.113
Month 18	54 (26–128)	53 (26–105)	0.48
Proteinuria, g/24 h			
Month 3	0.16 (0.00–2.18)	0.25 (0.00–2.00)	0.007
Month 6	0.15 (0.00–5.00)	0.18 (0.00–1.92)	0.69
Month 12	0.15 (0.00–2.26)	0.20 (0.00–3.90)	0.19
Month 18	0.16 (0.00–10.70)	0.16 (0.00–1.89)	0.79

Values are shown as median (range).

^a^Student's *t*-test or Wilcoxon test.

^b^aMDRD formula.

**Table 4:** Histological results of surveillance biopsies at one year posttransplant (central reading) among adequate samples (*n* = 116).

Histological lesions	Grade	*N* (%)
No significant changes (normal kidney graft)		51 (44)
Acute antibody-mediated rejection		0
Chronic antibody-mediated rejection		2 (1.8)
Acute cellular rejection	I B	2 (1.8)
Chronic cellular rejection		2 (1.8)
C4d+		3 (3)

Glomerulitis (g)	0	102 (87.9)
	1	8 (6.9)
	2	4 (3.4)
	3	2 (1.7)

Peritubular capillaritis (ptc)	0	98 (85.2)
	1	9 (7.8)
	2	7 (6.1)
	3	1 (0.9)

Acute tubular necrosis		12 (10.3)
IF/TA (Grade II and III)		35 (30.2)
CNI-related toxicity		15 (12.9)
Recurrence of initial disease		2 (1.7)
BK virus nephropathy		1 (0.9)
Thrombotic microangiopathy		3 (2.6)

IF/TA: interstitial fibrosis/tubular atrophy.

**Table 5:** Univariate and multivariate analysis of factors associated with IF/TA grade II or III on central biopsy reading for surveillance and diagnostic biopsies. All factors on univariate analysis showing an association (*P* < 0.1) are shown in the table and were included in the multivariate analysis.

Univariate analysis			
	IF/TA II or III (*N* = 38)	No IF/TA or IF/TA I (*N* = 86)	*P* value
Recipient gender, *n* (%)			
Male	17/71 (23.9)	54/71 (76.1)	0.06^a^
Female	21/53 (39.6)	32/53 (60.4)	
History of diabetes, *n* (%)			
Yes	6/8 (75.0)	2/8 (25.0)	0.010^b^
No	32/116 (27.6)	84/116 (72.4)	
Cold ischemia time, hours			
<12 hours	2/22 (9.1)	20/22 (90.9)	0.016^a^
≥12 hours	36/102 (35.3)	66/102 (64.7)	
Donor age, *n* (%)			
<60 years	22/86 (25.6)	64/86 (74.4)	0.066^a^
≥60 years	16/38 (42.1)	22/38 (57.9)	
Delayed graft function, *n* (%)			
Yes	13/27 (48.1)	14/27 (51.9)	0.026^a^
No	25/97 (25.8)	72/97 (74.2)	
Biopsy-proven acute rejection prior to biopsy, *n* (%)			
Yes	8/16 (50.0)	8/16 (50.0)	0.086^b^
No	30/108 (27.8)	78/108 (72.2)	
eGFR at month 3, mL/min/1.73 m^2 c^			
<50	22/47 (46.8)	25/47 (53.2)	0.002^a^
≥50	16/77 (20.8)	61/77 (79.2)	
eGFR at month 6, mL/min/1.73 m^2 c^			
<50	25/50 (50.0)	25/50 (50.0)	<0.001^a^
≥50	13/72 (18.1)	59/72 (81.9)	
Missing	0	2	

Multivariate analysis			
	Odds ratio	95% CI	*P* value

eGFR at month 6, mL/min/1.73 m^2 c^	3.85	1.64, 9.05	<0.002
<50 versus ≥50			
History of diabetes	0.17	0.029; 0.98	0.05
No versus Yes			
Cold ischemia time	0.18	0.036; 0.93	0.04
<12 h versus ≥12 h			

IF/TA: interstitial fibrosis/tubular atrophy.

^a^Chi squared.

^b^Fisher.

^c^aMDRD formula.

Percentages are calculated based on the denominator in each row.
